# The Role of Crowded Physiological Environments in Prion and Prion-like Protein Aggregation

**DOI:** 10.3390/ijms141121339

**Published:** 2013-10-28

**Authors:** Qian Ma, Ji-Ying Hu, Jie Chen, Yi Liang

**Affiliations:** State Key Laboratory of Virology, College of Life Sciences, Wuhan University, Wuhan 430072, China; E-Mails: mq@whu.edu.cn (Q.M.); jiji@whu.edu.cn (J.-Y.H.); chenjie@whu.edu.cn (J.C.)

**Keywords:** prion protein, Tau protein, prion- like protein, protein aggregation, macromolecular crowding, neurodegenerative diseases

## Abstract

Prion diseases and prion- like protein misfolding diseases are related to the accumulation of abnormal aggregates of the normal host proteins including prion proteins and Tau protein. These proteins possess self-templating and transmissible characteristics. The crowded physiological environments where the aggregation of these amyloidogenic proteins takes place can be imitated *in vitro* by the addition of macromolecular crowding agents such as inert polysaccharides. In this review, we summarize the aggregation of prion proteins in crowded physiological environments and discuss the role of macromolecular crowding in prion protein aggregation. We also summarize the aggregation of prion- like proteins including human Tau protein, human α-synuclein, and human copper, zinc superoxide dismutase under macromolecular crowding environments and discuss the role of macromolecular crowding in prion- like protein aggregation. The excluded-volume effects caused by macromolecular crowding could accelerate the aggregation of neurodegenerative disease-associated proteins while inhibiting the aggregation of the proteins that are not neurodegenerative disease-associated.

## 1. Introduction

Natively folded and functional proteins are the most important executors in a series of biological processes ranging from the synthesis of all kinds of biological molecules to cellular signal transductions and biochemical reactions. In a living cell, the cellular environment contains many volume-excluding macromolecules such as proteins, polysaccharides, and lipids that make the cellular environment very crowded [1–3]. During folding and processing in specific compartments after being synthesized in cells, incompletely folded proteins will inevitably be exposed to the solvent and interact with other molecules inappropriately and those off-pathway partially folded proteins could either be saved by cellular quality control mechanisms including molecular chaperones or degraded by cellular quality control mechanisms including proteasomes [4,5]. Should they escape such cellular quality control mechanisms, they will form aggregates with abnormal conformations [6]. So far, many neurodegenerative diseases have been found to be associated with the aggregation of specific proteins, and amyloid deposits of different proteins have been found in the brain of the patients such as prion diseases, Alzheimer disease, Parkinson disease, and amyotrophic lateral sclerosis (ALS) [6–10]. Such prion diseases and prion-like protein misfolding diseases are related to the accumulation of abnormal aggregates of the normal host proteins including prion proteins and Tau protein that possess self-templating and transmissible characteristics. As a result, studies of prion and prion-like protein misfolding both *in vivo* and *in vitro* are of great value in elucidating the development of neurodegenerative diseases. As neurodegenerative diseases are related to the self-association and fibrillization of amyloidogenic proteins, it is necessary to imitate crowded physiological environments where aggregation of such proteins takes place by the addition of macromolecular crowding agents *in vitro* [11–22].

### 1.1. Protein Aggregation *in vitro*

In order to make the conversion of proteins into amyloid fibrils *in vitro* possible, specific treatments have to be made because protein aggregation results from the association of partially folded chains rather than native proteins, for example, through the addition of polyanions or denaturants, and elevating temperatures or lowering pH [11]. Heparin, for instance, has been widely used to induce the aggregation of Tau protein efficiently [13,14,23–34]. Prion protein aggregation *in vitro* is usually promoted by denaturing agents such as urea and guanidine hydrochloride [13–15,35,36], and the aggregation of lysozyme is performed at pH 2.0, a very acidic environment [14,37,38].

### 1.2. Macromolecular Crowding and Protein Aggregation *in vitro*

The aggregation of proteins is a specific process and the effects of macromolecular crowding on it can be very complex. A multitude of effects of macromolecular crowding, including the excluded-volume effects of crowders, non-specific interactions between proteins and crowders, and increased viscosities and reduced diffusion constants by crowders, have to be taken into account [14,16,39–44]. For macromolecular crowding experiments choosing the right crowders is the key event. The crowders chosen should be very stable and not interact with the target proteins. Ficoll 70 and dextran 70 are the most widely used polysaccharide crowders [1,13–15,17,18], and bovine serum albumin (BSA) and hen egg white lysozyme are the most widely used protein crowders [20,21,37,44–49]. According to recent studies [14,16,39–45], effects of macromolecular crowders on protein association and aggregation are protein-dependent and crowder-dependent, and appear to be the balanced result of steric exclusion and non-specific interactions between proteins and crowders. In general, the excluded-volume effects of crowders seem to be predominant when polysaccharide crowders non- interaction with target proteins are used, whereas non-specific interactions between proteins and crowders seem to be predominant when protein crowders are used. The magnitude of such effects of macromolecular crowding is dependent on the physical and chemical properties of the proteins involved and the macromolecules that give rise to macromolecular crowding [14,16,39–45]. In two recent studies, however, the chaperone- like activity of BSA has been characterized at physiological concentrations, confirming the anti-aggregatory and anti-amyloid properties of BSA, and evidence has been provided that these properties arise from specific interactions between BSA and its target proteins [44,45]. Therefore, BSA could be a good choice for studying non-specific interactions between proteins and crowders and/or chaperone- like activity in the prevention of protein aggregation.

## 2. The Role of Crowded Physiological Environments in Prion Protein Aggregation

### 2.1. Human Prion Protein and Its Pathological Mutants

**Figure 1:**
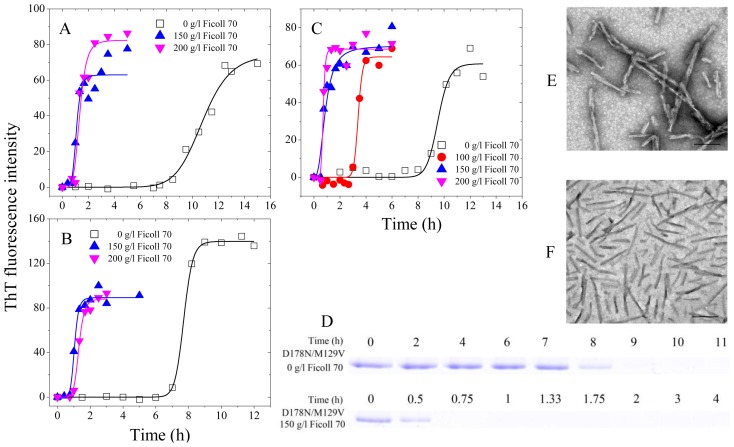
Macromolecular crowding enhances fibril formation of the recombinant human prion protein double mutants E196K/E219K (**A**), Q217R/E219K (**B**), and D178N/M129V (**C**–**F**). Fibril formation of pathogenic mutants in the absence and in the presence of Ficoll 70, monitored by ThT fluorescence (**A**–**C**) and Sarkosyl-soluble SDS-PAGE (**D**). In ThT fluorescence assay (**A**–**C**), 10 μM human PrP mutant was incubated at 37 °C in PBS buffer (pH 7.0) containing 2.0 M guanidine hydrochloride in the absence and presence of a crowding agent with continuous shaking at 220 rpm. The empirical Hill equation was fitted to the data and the solid lines represented the best fit. The crowding agent concentrations were 0 (**open square**), 100 g/L (**solid circle**), 150 g/L (**solid triangle**), and 200 g/L (**inverted solid triangle**), respectively. The assays were carried out at 37 °C. In Sarkosyl-soluble SDS-PAGE experiments (**D**), 20 μM D178N/M129V was incubated under the same condition described above. Samples were taken and dialyzed against 20 mM sodium acetate buffer, and incubated with 100 mM Tris-HCl buffer containing 2% Sarkosyl for 30 min. Then the samples were centrifuged at 17,000 *g* for 30 min and the supernatants were mixed with 2× loading buffer and separated by 13.5% SDS-PAGE. Gels were stained with Coomassie Blue R250. Transmission electron micrographs of D178N/M129V were made after incubation under different conditions (**E** and **F**). D178N/M129V samples were incubated for 11 h (**E**) or 2 h (**F**) in the absence of a crowding agent (**E**) and in the presence of 150 g/L Ficoll 70 (**F**), respectively. A 2% (*w/v*) uranyl acetate solution was used to negatively stain the fibrils. The scale bars represent 200 nm.

The misfolding of cellular prion protein PrP^C^ to its pathogenic conformation PrP^Sc^ is the key procedure for prion diseases including Creutzfeldt-Jakob disease (CJD) in humans, bovine spongiform encephalopathy (BSE) in cattle, and scrapie in sheep [8,13–15]. The conversion of prion protein (PrP) from a normal soluble conformation PrP^C^ to PrP^Sc^ is believed to occur on the cell surface, in the endocytic vesicles, or in the crowded extracellular matrix [13–15]. Authentic prion protein (PrP^C^) undergoes several posttranslational modifications including the addition of two *N*-linked glycosylations at amino acid residue position Asn-181 and Asn-197 [50,51] and a *C*-terminal glycosylphosphatidylinositol (GPI) anchor [51,52], but the recombinant PrP expressed in *Escherichia coli* does not undergo such posttranslational modifications. The Baskakov lab [35] has provided the first demonstration that full- length recombinant PrP with an intact disulfide bond at physiological concentrations can be converted into amyloid conformation *in vitro*. The same group has revealed that in dilute solutions, fibrils formed by recombinant PrP are intrinsically promiscuous and capable of utilizing heterologous PrP variants as a substrate in a highly efficient manner [36]. We have used polysaccharide crowding agents to mimic the crowded extracellular matrix, and investigated the misfolding of disease-associated human PrP and its pathological mutants and bovine PrP in such crowded physiological environments [13–15]. We have reported that such crowded cell- like environments dramatically promote fibril formation of the recombinant human PrP and bovine PrP [13,15] and significantly accelerate the nucleation step of fibril formation of the recombinant human PrP and its two pathogenic mutants, CJD-associated E196K and fatal familial insomnia-associated D178N [14]. Furthermore, macromolecular crowding causes the recombinant human PrP to form short fibrils and nonfibrillar particles with lower conformational stability and higher protease resistance activity, compared with those formed in dilute solutions [13]. Our data has demonstrated that a crowded physiological environment could play an important role in the pathogenesis of prion diseases by accelerating amyloidogenic PrP misfolding and inducing human PrP fibril fragmentation, which is considered to be an essential step in prion replication [13]. Figure 1 contains our previously unpublished data. The methods for expression, purification, and fibrillization of three human PrP double mutants were the same as those previously described [14]. All kinetic experiments were repeated three times. As shown in Figure 1A and 1B, the presence of Ficoll 70 at concentrations of 150–200 g/L in the reaction systems significantly accelerated amyloid formation of the recombinant human PrP double mutants, CJD-associated E196K/E219K and Q217R/E219K, on the investigated time scale. Moreover, the enhancing effect of Ficoll 70 on fibril formation of these two double mutants was stronger than that of the wild-type human PrP (Figure 1 and [14]). Similarly, the presence of Ficoll 70 at 100–200 g/L in the reaction systems also significantly accelerated fibril formation of the recombinant human PrP double mutant D178N/M129V on the investigated time scale (Figure 1C). As shown in Figure 1D, a clear band corresponding to Sarkosyl-soluble human PrP monomers was observed when CJD-associated D178N/M129V was incubated in the absence of a crowding agent for 8 h, while the human PrP monomer band was observed when D178N/M129V was incubated with 150 g/L Ficoll 70 for a much shorter time (0.5 h). Furthermore, the enhancing effect of Ficoll 70 on fibril formation of CJD-associated D178N/M129V was stronger than that of fatal familial insomnia-associated D178N (Figure 1 and [14]). Clearly, the presence of a strong crowding agent dramatically promoted amyloid fibril formation of three pathological human PrP double mutants, CJD-associated E196K/E219K, Q217R/E219K, and D178N/M129V (Figure 1). Transmission electron microscopy was employed to characterize the morphology of human PrP aggregates formed in the absence and in the presence of crowding agents. The addition of 150 g/L Ficoll 70 had no significant effect on the morphology of the recombinant human PrP mutant D178N/M129V samples, and many straight fibrils with a length of 100–500 nm were observed in these samples (Figure 1E,F). Figure 1 represents the general idea that the excluded-volume effects caused by macromolecular crowding could strongly accelerate the nucleation step of fibril formation of amyloidogenic proteins, such as human PrP. It should be pointed out that the differences between 150 and 200 g/L are relatively low and do not appear significant. The work of Huang *et al.* [53] has shown that macromolecular crowding as a potential intracellular factor could convert the recombinant human PrP^C^ to the soluble neurotoxic β-oligomers. Recently, Bergasa-Caceres and Rabitz [54] have developed a model to quantitatively study the effect of macromolecular crowding, and found that macromolecular crowding increased both the stability and the folding rate of murine prion protein fragment.

### 2.2. Rabbit Prion Protein

Rabbits are among the few mammalian species that exhibit resistance to prion diseases [14,15,55]. In order to understand the molecular mechanism underlying the resistance of rabbits to prion diseases, we have used polysaccharide crowding agents to mimic the crowded extracellular matrix, and investigated and compared the effects of macromolecular crowding on the misfolding of non-disease associated rabbit PrP and disease-associated human/bovine PrPs [14,15]. We have reported that such crowded environments significantly inhibit fibrillization of the rabbit PrP by stabilizing its native state [14,15]. Furthermore, amyloid fibrils formed by the rabbit protein do not generate a proteinase K (PK)-resistant fragment of 15–16 kDa, but those formed by the proteins from human and cow generate such PK-resistant fragments [15]. We thus suggest that the strong inhibition of fibrillization of the rabbit PrP by the crowded physiological environment and the absence of such a protease-resistant fragment for the rabbit PrP could be two of the reasons why rabbits are resistant to prion diseases [15].

## 3. The Role of Crowded Physiological Environments in Prion-like Protein Aggregation

Several recent experimental findings and clinical observations have suggested that protein aggregates associated with Parkinson, Alzheimer, and Huntington diseases could move from cell to cell and spread from affected to unaffected areas of the brain, suggesting prion- like transmission in these neurodegenerative diseases [56–58]. Such transmission and spreading processes take place in crowded physiological environments. In this review, we summarize the aggregation of prion- like proteins including human Tau protein, human α-synuclein, and human copper, zinc superoxide dismutase (SOD1) under macromolecular crowding environments and discuss the role of macromolecular crowding in prion-like protein aggregation.

### 3.1. Tau Protein

The intracellular aggregates of microtubule-associated protein Tau called neurofibrillary tangles are one of the important hallmarks of Alzheimer disease [5,9]. In other words, the misfolding of Tau in human brain is one of the key processes in the development of Alzheimer disease. Recent reports have suggested that Tau fibrils can seed fibril formation of intracellular Tau both in adjacent cells and transgenic mice [58]. The misfolding of Tau protein has been widely investigated in dilute solutions [23–34]. Goedert and co-workers [28] have provided the first demonstration that recombinant Tau protein forms paired helical- like filaments under physiological conditions *in vitro*, when incubated with heparin or heparan sulphate. Following our previous study of quantitative characterization of heparin- induced fibril formation of Tau protein [24], the Udgaonkar lab [32] has demonstrated that heparin plays a role in only the nucleation steps and not the elongation steps of Tau aggregation *in vitro*. Very recently, Elbaum-Garfinkle and Rhoades [34] have observed that heparin binding induces a distinct two-state structural transition in Tau protein characterized by a loss of long-range contacts and accompanied by a compaction of the microtubule-binding domain of Tau. Tau aggregation in crowded physiological environments has been characterized in our lab [13,14]. We have demonstrated that macromolecular crowding enhances the fibrillization of both human Tau fragment Tau_244–372_ and glycogen synthase kinase-3β (GSK-3β) hyperphosphorylated Tau_244–441_[13,14]; while the latter does not form filaments in the absence of a crowding agent on the investigated time scale, it does form fibrils in the presence of a crowding agent [14]. Furthermore, crowded cell- like environments significantly accelerate the nucleation step of fibril formation of hyperphosphorylated Tau_244–441_[13]. We thus suggest a compensation mechanism of macromolecular crowding for the lost capability of fibril formation caused by the hyperphosphorylation of Tau [13].

### 3.2. α-Synuclein

The fibrillization and aggregation of a presynaptic protein α-synuclein is a key process involved in the formation of cytoplasmic inclusions called Lewy bodies in substantia nigral neurons and, potentially, in the pathology of Parkinson disease [7]. Like Tau protein, human α-synuclein is also intrinsically disordered. Considerable work has shown that crowding agents stimulate amyloid fibril formation of α-synuclein *in vitro* [19–22,59]. Shtilerman *et al.* [19] have reported that three crowding agents, Ficoll 70, dextran 70, and high molecular weight polyethylene glycol (PEG), increase the effective concentration of α-synuclein and reduce the lag time for protofibril and fibril formation without altering their morphology. Uversky and co-workers [20,21] have found the addition of high concentrations of different crowders (inert proteins, polysaccharides, and neutral PEGs) dramatically accelerates α-synuclein fibrillation *in vitro*, and that the magnitude of the excluded-volume effect depends on the nature of the crowder, its length and concentration. Uversky and colleagues [22] have further analyzed the effect of macromolecular crowding on fibrillization of four proteins, and concluded that fibrillization of monomeric natively unfolded proteins such as human α-synuclein is accelerated by macromolecular crowding but fibrillization of oligomeric proteins such as human insulin in crowding environment is slowed down. One year later, the same group suggested that the fibril-promoting effects of factors inducing partial folding of α-synuclein and the fibril- stimulating effects of macromolecular crowding are relatively independent and thus might act additively [59].

### 3.3. Copper, Zinc Superoxide Dismutase (SOD1)

ALS, partly caused by the mutations and aggregation of human SOD1, is a fatal degenerative disease of motor neurons [10]. Pathological human SOD1 mutant A4V is the most common familial ALS mutation in North America and is associated with a very rapid disease progression [14,60,61]. We have investigated the effects of macromolecular crowding on amyloid fibril formation of such a pathogenic mutant, and found that crowded cell- like environments significantly accelerate the nucleation step of fibril formation of A4V SOD1 [14]. Pathological mutant A4V is a folded protein with reduced stability [62], so that macromolecular crowding enhances aggregation of such a not-particularly-stable protein more than folding [14].

### 3.4. Amyloid-β

One of the important hallmarks of Alzheimer disease is the accumulation of insoluble fibrils in the brain composed of amyloid-β (Aβ) peptides with parallel in-register cross-β-sheet structure [17,63]. In other words, the misfolding of Aβ in the brain of human is one of the key procedures for Alzheimer disease. Considerable work has shown that macromolecular crowding stimulates misfolding of Aβ *in vitro* [17,63,64]. It has been demonstrated that macromolecular crowding significantly enhances the rate of amyloid formation of Aβ_1–40_ at membrane surfaces, which can be explained by the excluded-volume effect of a crowder, increasing the effective concentration of Aβ monomers in solution and forcing Aβ toward the membrane surface [64]. Recently, Yeung and Axelsen [63] reported that monomeric Aβ_1–40_ peptides form extended β-strand seed structures for nucleating amyloid fibril formation in a crowded macromolecular environment mimicked by a reverse micelle. In the same year, Lee and co-workers [17] investigated the effects of two polysaccharide crowders (dextran and Ficoll) and two different experimental conditions (with and without shaking) on the fibrilization of Aβ_1–40_ peptides. They have demonstrated that with shaking, both dextran and Ficoll accelerate Aβ nucleation, but without shaking, viscosity dominates over the excluded-volume effect of a crowder [17]. In case of Aβ in Alzheimer disease patients, it is assumed that soluble Aβ oligomers exhibit the main toxicity but not the Aβ fibrils [65], and thus a crowded physiological environment could play a neuroprotective role in the onset and progression of Alzheimer disease by inducing the most toxic Aβ oligomers to form innocuous Aβ fibrils.

### 3.5. Other Proteins

Considerable work about the effects of macromolecular crowding on the folding and misfolding of proteins has been done [1–3,11] and crowded physiological environments play an important role in cell biology [66]. Macromolecular crowding can accelerate folding steps that involve structural collapse and decelerate folding steps that involve local unfolding of intermediates [67]. We have demonstrated that mixed macromolecular crowding accelerates the refolding of hen egg white lysozyme and rabbit muscle creatine kinase [46,68] but inhibits amyloid formation of hen egg white lysozyme by stabilizing its native state [37]. Engel and colleagues [67] demonstrated that macromolecular crowding destabilizes unfolded apoflavodoxin, which becomes more compact, and causes severe aggregation of the off-pathway intermediate of apoflavodoxin. Minton and co-workers [69] have investigated the effects of macromolecular crowding on amyloid formation of human apolipoprotein C-II, and found that the rate and extent of amyloid formation by this protein is substantially enhanced by the addition of a polysaccharide crowder, dextran T10. Furthermore, the observed dependence of the overall rate of amyloid formation on dextran concentration could be accounted for quantitatively by a simple model for non-specific volume exclusion [69]. Interestingly, in *Escherichia coli*, the localization of aggregates of misfolded proteins at the poles or in the center of the cell has been recently linked to aging, which is due to the coupling of passive diffusion-aggregation with the spatially non-homogeneous macromolecular crowding [70]. Recently, Milles and colleagues [71] have studied the aggregation behavior of intrinsically disordered and phenylalanine-glycine-rich nucleoporins (FG Nups) under molecular crowding conditions that mimic the interior of the nuclear pore complexes, and found that molecular crowders can accelerate the aggregation and amyloid formation speed of yeast and human FG Nups by orders of magnitude.

## 4. Conclusions

**Figure 2:**
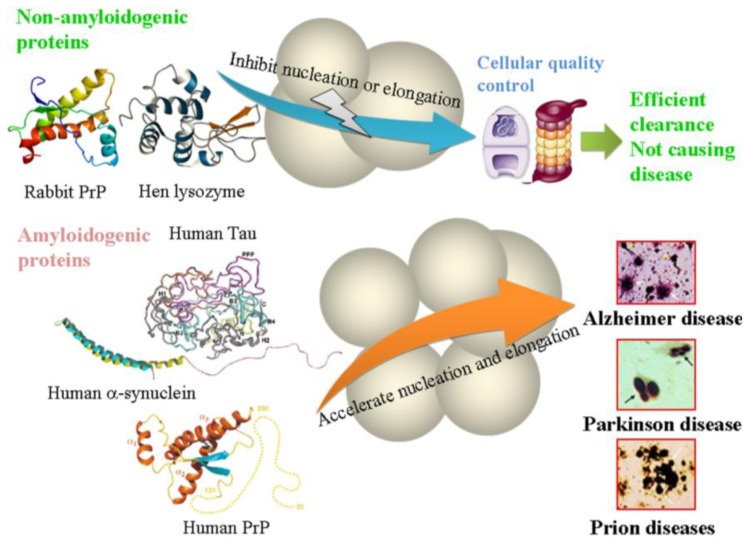
Regulatory mechanism of crowded physiological environments. The excluded-volume effects caused by macromolecular crowding could accelerate the aggregation of neurodegenerative disease-associated proteins while inhibiting the aggregation of the proteins that are not neurodegenerative disease-associated. The images of Alzheimer, Parkinson, and Prion disease aggregates are adapted from Figure 1 with permission from [72]. Copyright Nature Publishing Group, 2003.

Amyloidogenic proteins are proteins associated with neurodegenerative disease, and non-amyloidogenic proteins are proteins that are not neurodegenerative disease-associated [14]. Prion diseases and prion- like protein misfolding diseases are resulted from the accumulation of abnormal aggregates of amyloidogenic proteins such as prion protein and Tau protein with self-templating and transmissible characteristics. All proteins reported on here are widely studied. The crowded physiological environments where the aggregation of such amyloidogenic proteins takes place can be mimicked experimentally by adding a high concentration of a non-specific crowding agent into the system *in vitro*. In this review, we summarize the aggregation of PrPs from different mammalian species in crowded physiological environments. Macromolecular crowding has been found to inhibit fibrillization of the rabbit PrP significantly but promote fibril formation of the human and bovine PrPs dramatically and enhance the folding of the murine PrP [13–15,53,54]. The strong inhibition of fibrillization of the rabbit PrP by the crowded physiological environment and the difference in structure and the PK-resistant feature between amyloid fibrils formed by the rabbit PrP and those formed by the proteins from human and cow might be two of the reasons why rabbits are resistant to prion diseases [14,15]. In addition, aggregation of hen egg white lysozyme is also strongly inhibited by macromolecular crowding [14,37]. We also summarize the aggregation of prion- like proteins, such as human Tau protein, human α-synuclein, human SOD1, and human Aβ, in crowded physiological environments. In general, a crowded physiological environment could play an important role in the pathogenesis of Alzheimer disease by accelerating Tau protein misfolding [13,14] and Aβ misfolding [17,63,64], in the pathogenesis of prion diseases by accelerating amyloidogenic PrP oligomerization and misfolding [13–15,53,54] and inducing human PrP fibril fragmentation [13], in the pathogenesis of Parkinson disease by accelerating α-synuclein misfolding [19–22,59], and in the pathogenesis of ALS by accelerating SOD1 misfolding [14]. Obviously, as illustrated in Figure 2, the excluded-volume effects caused by macromolecular crowding could accelerate the nucleation and elongation steps of fibril formation of amyloidogenic proteins, such as human PrP, human Tau and human α-synuclein [72], while inhibit the nucleation or elongation step of fibril formation of non-amyloidogenic proteins, such as rabbit PrP and hen lysozyme [14]. We suggest that amyloidogenic proteins are more likely to form amyloid fibrils under crowded conditions than in dilute solutions. By contrast, non-amyloidogenic proteins are unlikely to misfold in crowded physiological environments. For non-amyloidogenic proteins, the resulting molecules are efficiently cleared by cellular quality control mechanisms in combination with the strong inhibition of fibrillization of such proteins by the crowded physiological environment, thereby not causing any neurodegenerative diseases (Figure 2). It should be pointed out that according to our definition, FG Nups are not amyloidogenic proteins but their aggregation is dramatically accelerated under molecular crowding conditions [71]. This unusual case provides some insight into how such amyloids can grow into networks to adapt the macroscopic phenotype of a hydrogel [71]. Therefore crowded physiological environments could play an important role in the cellular quality control mechanisms by regulating protein folding and aggregation in cells. Information presented here clarifies how amyloidogenic proteins misfold and how non-amyloidogenic proteins avoid misfolding in crowded physiological environments which will lead to a better understanding of the molecular mechanisms of neurodegenerative diseases.
